# Right Ventricular free wall myocardial tissue characterisation by systolic Cardiac Magnetic Resonance T1 mapping in pulmonary hypertension

**DOI:** 10.1186/1532-429X-18-S1-P147

**Published:** 2016-01-27

**Authors:** Geeshath Jayasekera, Colin Church, Martin Johnson, Andrew Peacock, Aleksandra Radjenovic

**Affiliations:** 1 Golden Jubilee National Hospital, Glasgow, Glasgow, UK; 2 Institute of Cardiovascular and Medical Sciences, University of Glasgow, Glasgow, UK

## Background

Pulmonary hypertension is a rare progressive disorder characterised by elevated pulmonary artery pressure leading to right ventricular (RV) failure and death. Cardiac MR (CMR) derived right and left ventricular functional variables have shown to be prognostic in pulmonary hypertension. Native T1 mapping is a CMR technique of myocardial tissue characterisation without the need for a reference area or contrast administration. T1 mapping of the RV free wall has shown to have poor inter-observer reproducibility due to the relatively thin RV myocardium. An alternative is to acquire T1 maps in systole when the RV myocardium is thicker. We investigated whether native T1 values in systole relate to invasive pressure measurements and markers of RV dysfunction in patients with pre-capillary pulmonary hypertension (PH).

## Methods

Twenty pre-capillary pulmonary hypertension patients (mean pulmonary artery pressure 46.8 ± 10.6 mmHg) (16 WHO group 1, 4 WHO group 4) underwent cardiac MR and right heart catheterisation. Ventricular volumes and ejection fraction were determined by CMR while pulmonary artery pressure measurements and cardiac output were obtained during right heart catheterisation. T1 maps were acquired on a Siemens Avanto 1.5T scanner using a MOLLI sequence on a mid ventricular short axis plane with a trigger delay to coincide with systole. Native T1 values were determined using regions of interest (ROI) in the RV free wall, Inter-ventricular septum (IVS) and left ventricle (LV). RV insertion points were excluded. ROI size and placement ensured avoidance of partial volume effects, especially in the RV free wall.

## Results

The Native T1 values of the RV free wall were significantly higher compared to LV (p < 0.001) and IVS (p < 0.001). There was no significant difference between T1 values of the LV and IVS. Native T1 values of the RV were related to RV ejection fraction (p < 0.01) and RV end-systolic volume (p < 0.005) but did not correlate with right heart catheter derived invasive pressure measurements.

## Conclusions

The inter-ventricular septum adapt differently to high RV pressures compared to the RV free wall. The native T1 values of the RV free wall are increased in patients with pre-capillary PH and are associated with CMR markers of RV dysfunction. These may suggest myocardial histological changes. Native T1 imaging may become a potential biomarker in patients with pulmonary hypertension.

**Figure 1:**
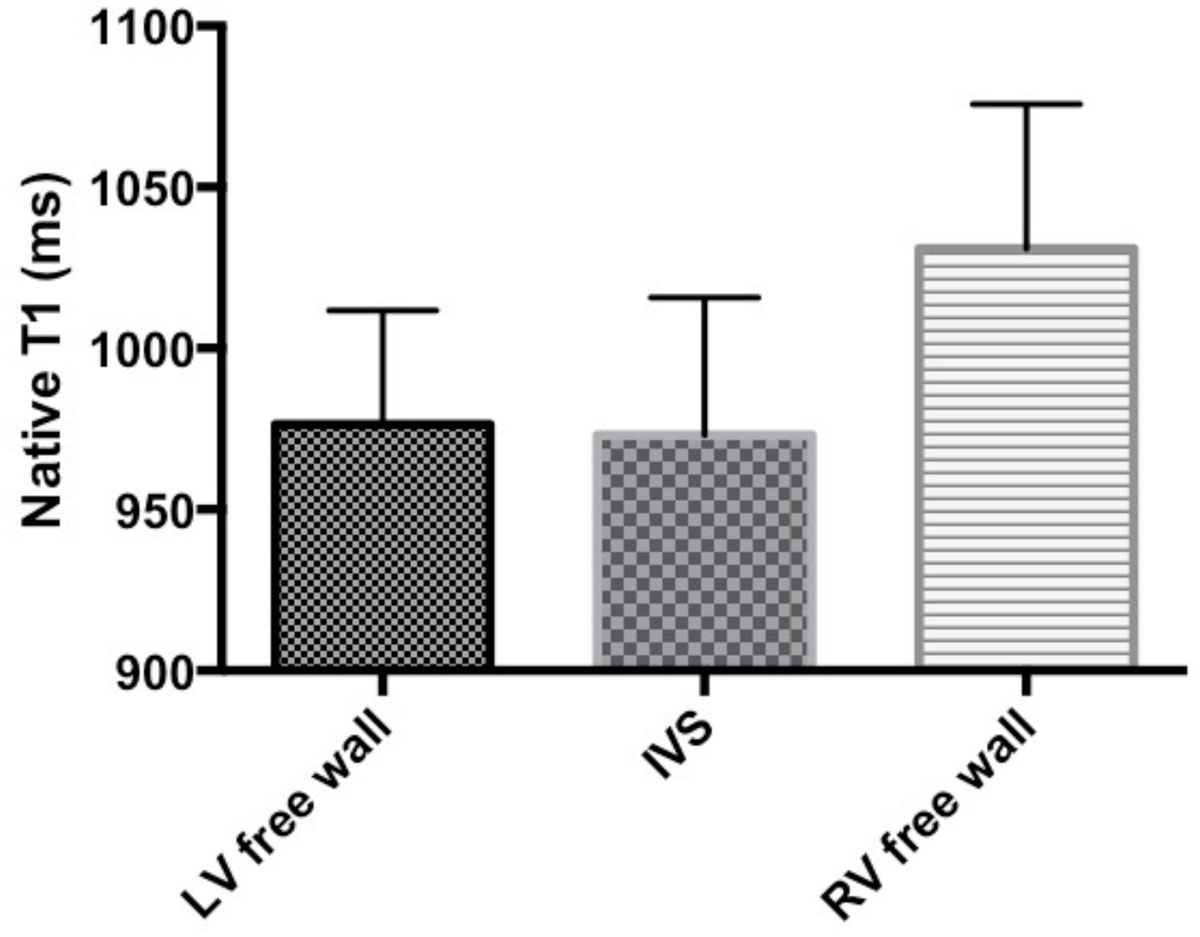
**T1 values of LV free wall vs Interventricular septum vs RV free wall in patients with Pulmonary Hypertension**.

